# A Mind–Body Approach to Pediatric Pain Management

**DOI:** 10.3390/children4060050

**Published:** 2017-06-20

**Authors:** Melanie L. Brown, Enrique Rojas, Suzanne Gouda

**Affiliations:** 1 Department of Pediatrics, The University of Chicago, Chicago, IL 60637, USA erojas@peds.bsd.uchicago.edu (E.R.); Suzanne.Gouda@uchospitals.edu (S.G.); 2 Department of Pain, Palliative Care and Integrative Medicine, Children’s Hospitals and Clinics of Minnesota, Minneapolis, MN 55404, USA

**Keywords:** mind–body medicine, pain management, pediatrics, acupuncture, yoga, meditation, hypnosis

## Abstract

Pain is a significant public health problem that affects all populations and has significant financial, physical and psychological impact. Opioid medications, once the mainstay of pain therapy across the spectrum, can be associated with significant morbidity and mortality. Centers for Disease and Control (CDC) guidelines recommend that non-opioid pain medications are preferred for chronic pain outside of certain indications (cancer, palliative and end of life care). Mindfulness, hypnosis, acupuncture and yoga are four examples of mind–body techniques that are often used in the adult population for pain and symptom management. In addition to providing significant pain relief, several studies have reported reduced use of opioid medications when mind–body therapies are implemented. Mind–body medicine is another approach that can be used in children with both acute and chronic pain to improve pain management and quality of life.

## 1. Introduction

Pain is a significant public health problem leading to lost days of school and increased use of the healthcare system. Pain affects all populations and can significantly impact quality of life [1]. Over 11 million children have special health care needs and about 60% of them have difficulty participating in any activity [2]. In one cross-sectional study of pain prevalence in a pediatric hospital, the authors found that out of 241 patients, 27% had pain at the time of admission and 77% experienced pain at some point during their admission; what is more, pain medication was found to be single-agent and administered irregularly [3]. 

In recent years, opioid diversion, as well as the overuse and over prescription of opioid medications, have come to the forefront as a significant public health concern. Integrative medicine uses a patient centered approach to combine conventional medicine with evidence-based complementary approaches. Integrative therapies such as mind–body medicine can provide a non-opioid and nontoxic approach to pain management across the spectrum. Even in the case of end-of-life care [4,5], integrative and mind–body therapies are recommended [4,5]. For example, the Hospice and Palliative Nurses Association’s most recent position statement supports and encourages the competent practice of complementary therapies for the purpose of promoting holistic end-of-life-care.

## 2. The Problem

### 2.1. What Is Pain

Pain can be defined as an unpleasant sensation. In some cases, pain is a warning sign of actual or potential tissue damage. However, dysregulation of the nervous system can also lead to a pain sensation when there is no present danger [6,7]. In addition to the physical sensation, pain behaviors are also influenced by pain perception. In a recent randomized control trial, pain sensitivity was assessed in over 700 adult patients with major depression. It was found that those suffering from major depression had higher pain sensitivity even after adjusting for factors such as poor sleep and physical inactivity [8]. Severity of anxiety also predicted decreased pain threshold. 

Chronic pain is known to be associated with changes in not only brain function, but also in brain structure. In 2013, researchers demonstrated that the brain areas that were activated in acute low back pain for example were limited to regions involving acute pain; in the chronic pain group, activity was found in the emotion-related circuitry of the brain [9]. 

Pain perception and experience in the pediatric population is complex and multi-faceted, including but not limited to the nidus of pain itself, the fear of pain, previously developed pain memories, and familial influence. Acute pain is usually classified as pain lasting less than three months with a sudden onset that is often related to tissue damage. When considering the development of chronic pain in pediatrics, typically defined as pain persisting beyond three months or beyond the expected duration of healing, a fear-avoidance response can emerge, leading to a self-fulfilling cycle made up of pain, emotional distress, and functional disability [10]. In pediatrics, there is also an added complexity when considering developmental stage and parental influence. Not only do parents often have to serve as the surrogate communicator for the patient, parents own magnification, rumination, and anxiety surrounding pain influences the child and vice versa, leading to increased pain sensitivity and fear-avoidance behaviors [11]. It is clear that pain is not as simple as a noxious stimulus to an extremity that sends a danger signal to the brain.

### 2.2. The Opioid Crisis

Increases in opioid prescriptions have been noted over the past decade [12,13,14]. The opioid overdose and death rates have also been noted to increase [15,16,17,18,19,20]. 2016 Centers for Disease and Control (CDC) guidelines state that that non-opioid therapy is preferred for chronic pain outside of active cancer, palliative and end-of-life care in patients over the age of 18 years. Inappropriate medication use and dosing can lead to death and disability [15,16,17,18,19,20]. At times, significant morbidity can result even when recommended dosage ranges are used [15,16,17,18,19,20]. For example, deaths have been described with the use of codeine despite dosages prescribed in the recommended range. In the case of codeine, the prodrug must be converted into morphine in the liver via the cytochrome P450 2D6 system and drug over or under conversion may be the result of genetic variation [21]. Furthermore, tolerance, withdrawal, and dependence present a problem even in the pediatric population and the neuropsychological effects of opioids may also be a cause for concern [22,23,24,25]. Opioid medications can play an important role in the treatment of pain; however, a pain management plan is not complete without including mind–body therapies.

### 2.3. Pain in Pediatrics

In pediatrics, pain, both acute and chronic, is often under-recognized and under-treated [26]. Studies have shown that up to 40% of children experience pain at least weekly, and conservative estimates say chronic pain affects 20% to 35% of children and adolescents around the globe [27]. Despite this significant prevalence, pain often goes under-addressed. A large-scale study involving eight pediatric hospitals observed inadequate pain assessment and management for patients undergoing painful procedures, reporting that less than one-third of patients had documentation of one or more pain management interventions [28]. When pain becomes chronic, significant physical and psychological tolls are experienced by both the patient and their families. Often the pain itself continues into adulthood, with 17% of adult chronic pain patients reporting a history of chronic pain in childhood/adolescence [29]. In addition, patients with chronic pain are at increased risk for several comorbidities, including many psychiatric disorders, hyperactivity disorders, social disability, and educational/occupational disability [10]. This patient population also has increased use of medical services, with healthcare costs for children with moderate to severe chronic pain averaging 19.5 billion annually [30]. Despite approximately 1.7 million children affected by chronic pain in the US alone with approximately 20% of cases having developed from acute post-operative pain, assessment and management of pain in pediatrics continues to be a challenge [26,31]. Mind–body medicine can provide a different approach. The mind–body approach is one in which the strengths of the patient and family are considered. In addition, this approach focuses on designing a treatment plan that is efficacious and minimizes the need for opioid medications. Mind–body remains a relatively novel approach in medicine. Although most of the current literature focuses on adult populations and on the more conventional psychological therapies (e.g.; cognitive behavioral therapy and dialectical behavioral therapy) that share similar inherent characteristics with mind–body approaches [32,33,34], there is a growing body of evidence that supports other mind–body therapies as effective and practical treatment approaches in pediatrics [35,36,37,38,39], particularly in the symptomatic treatment of cancer [4,40,41,42,43,44,45]. 

## 3. The Mind–Body Approach to Pain

### 3.1. The Use of Mind–Body Medicine in Pain Management

The American Academy of Pediatrics’ clinical report on Mind–Body Therapies in Children and Youth describes mind–body therapies as those that focus on the interaction between the mind and the body, with the intent to use the mind to influence physical functions and directly affect health [46]. Mind–body therapies show promise as adjunct and at times primary treatment for pain in children and adults, and can be used across the spectrum. For example, diaphragmatic breathing stimulates the vagus nerve and promotes the relaxation response. The vagus nerve, an essential component of the autonomic nervous system, affects many of the body’s internal organs including: the heart, lungs, liver, spleen, kidneys and gastrointestinal tract. As with many therapies, a development-based approach is essential. Teens can use complex imagery, while younger children can be taught diaphragmatic breathing techniques with simple imagery, such as blowing a balloon or blowing out candles. A toddler can be encouraged to engage in diaphragmatic breathing with the use of bubbles or pin wheels. For infants or children with severe cognitive disabilities, rocking and rhythmic womb or heartbeat sounds are techniques that can encourage diaphragmatic breathing. 

These techniques are well tolerated in children [36,44,47,48] and can be used in a developmentally appropriate manner to serve as adjunct or primary pain management [47,48]. Furthermore, a reduction in opioid use may be seen. One study demonstrated a greater than 60% reduction of opioid-like medication usage following routine surgery when acupuncture was used [49,50,51]. Although more research is needed in the pediatric population, given the low risk and low cost of many of these techniques [52,53,54] for patients and potentially for insurance companies as well, their use is encouraged to enhance symptom management whenever feasible. In the next section, we give an overview of selected mind–body therapies and their function and applications in the management of pain in the pediatric population.

### 3.2. Selected Mind–Body Approaches

#### 3.2.1 Meditation and Mindfulness 

Over the past few decades, mindfulness has emerged as a fundamental component of numerous therapies and interventions for a wide spectrum of clinical aliments [55]. Mindfulness, described as “the awareness that emerges through paying attention on purpose, in the present moment, and non-judgmentally to the unfolding of experience moment by moment”, is a meditation practice with ancient Buddhist origins that focuses on experiencing the present moment unobstructed by bias or judgmental thinking in an effort to improve cognitive and emotional well-being [56]. One such application of mindfulness is Kabat Zinn’s Mindfulness-Based Stress Reduction (MBSR), a group intervention first introduced in 1990 that focuses on mindfulness meditation training as a complimentary therapy to the standard medical treatment of chronic pain and illness [57,58,59,60,61,62,63,64]. Research has suggested that mindfulness can improve symptoms associated with medical illnesses and increase quality of life [65]. From a neuroscientific perspective, magnetic resonance imaging (MRI) and functional magnetic resonance imaging (fMRI) studies have been conducted in hopes of identifying the neural mechanisms that are responsible for the efficacy of mindfulness meditation in pain relief [66,67,68,69,70,71,72,73]. In one study, thirteen skilled Zen mediators, each having had a minimum of 1000 hours of meditation experience, were recruited and experimentally exposed to pain via thermal stimuli while in an MRI [70]. During exposure to pain, the meditators exhibited increased brain activation in the insula, thalamus, and midcingulate cortex; areas associated with the sensory aspect of pain. Additionally, decreases in brain activity were observed within the hippocampus, amygdala, and caudate; areas responsible for the recollection, emotion, and appraisal components of pain, respectively. The authors concluded that the participants were completely aware of the sensation of pain but were able to inhibit the appraisal and emotional responses of pain. In other words, changes in the perception of pain were facilitated through the cognitive and affective components of the pain matrix rather than through the sensory properties of pain. Furthermore, the differences in brain activity were found to be inversely proportional to meditation skill level, establishing a correlation that supports the authors’ hypothesis in regards to meditation’s therapeutic effect on pain. As for neurophysiological findings, structural MRI results overlapped with the fMRI results: meditators were found to have thicker grey-matter in the same pain-related regions of the brain where changes in functional activity were observed [66,68,69]. 

In terms of overall clinical outcome research, controlled trials of adults suffering from various forms of chronic pain (chronic low back pain, chronic headache/migraine, chronic neck pain, arthritis, cancer, and fibromyalgia) have indeed demonstrated improved pain ratings in regards to multiple dimensions of pain including intensity, acceptance, functional limitations, quality of life, and psychological well-being [62]. Nonetheless, mindfulness as it relates to pain in children has not been extensively studied and although mindfulness meditation has shown to be beneficial in classroom and school settings for improving psychological distress [74,75,76,77], more research is required in order to determine whether the same effects can be translated in children and pediatric medicine. 

#### 3.2.2. Hypnosis

Hypnosis as a form of therapy has a long history and has been widely used across various disciplines of health care. While the current research establishes hypnosis as a beneficial treatment for the management of pain in regards to both acute medical conditions, such as trauma and post-operative care, and chronic medical conditions, such as cancer and sickle cell anemia [78,79,80]; it is only since the 1980s that it has been meaningfully applied to pediatric care [81,82]. 

In general, hypnosis includes three phases: induction, suggestion, and emergence [82]. During induction, the provider encourages patient relaxation by asking them to imagine a calm and serene setting on which they can focus all of their attention. Next, the patient is given therapeutic suggestions to achieve the desired effect. Lastly, the patient is asked to leave their imagined setting and to return to normal consciousness. Hypnosis for pain management follows this same protocol with a focus on suggestions that either turn down or decrease pain perception or increase pain thresholds [83]. 

Given the significance of the suggestion stage of hypnosis, an important factor in clinical outcomes is the degree to which an individual is responsive or susceptible to hypnotic suggestions—a trait that is often referred to as hypnotizability. Of note, studies that have attempted to measure hypnotizability among children via the Children’s Hypnotic Susceptibility Scale and the Stanford Hypnotic Scale for Children have shown a positive correlation between hypnotizability [84,85] and age, thus suggesting that hypnosis may be an especially viable form of therapy for pain management in the pediatric population [82]. 

Taking a neuroscientific approach, neuro-imaging studies have attempted to measure the effects of hypnosis on the neuroanatomy and the neuro-cognitive functions of the brain in the context of pain [85,86,87,88]. In other words, many researchers have set out to investigate how hypnosis affects the brain’s neural-networks and physiology that in turn, are responsible for the perception of pain within an individual. This “pain matrix”, as it has been described, is comprised of specific areas of the brain that collectively produce the experience of pain. In the simplest summary of the current literature, the components of the pain matrix include: the prefrontal cortex, frontal lobes, anterior cingulate cortex, primary and secondary somatosensory cortices, thalamus and insula. The cerebellum, though not technically a component of the pain matrix, also plays a role. Using fMRIs to measure brain activity during hypnosis, researchers have concluded that by influencing activity in the various components of the pain matrix, hypnosis is indeed able to have a collective therapeutic effect on pain. 

Research focusing on clinical and experimental outcomes has also yielded positive results. A meta-analysis performed in 2000 of 18 studies found hypnosis to have a moderate to large analgesic effect [78]. When compared to groups receiving standard treatment and groups receiving no treatment, 75% of participants receiving hypnotic suggestion experienced a greater analgesic effect. Furthermore, the effect was seen with both clinical and experimental pain with no significant difference between the two settings. 

Hypnosis is a tool that carries minimal risk when used appropriately for pain management. It can be used by patients as well as practitioners. The goal is often to instruct the patient on the hypnotic technique so that hypnosis will become a tool of empowerment that the patient is able to use themselves at appropriate times for symptom management. It is important to note that hypnosis should only be performed by an appropriately trained practitioner and only to treat conditions that the practitioner is competent to treat. 

#### 3.2.3. Yoga

Yoga is another mind–body modality that should be considered in the management of pain. In brief, the ancient practice of yoga aims to unite the mind, body, and spirit through different isometric exercises, body poses, and mindful breathing. The goal is to optimize body functioning and reconditioning, skeletal realignment, and blood/lymph flow to the tissues [89]. Beyond the biophysical benefits, yoga is also inexpensive, can be practiced by people of any age and physical skill level, and can be performed almost anywhere. The side effects are minimal, with the most common being musculoskeletal injury, often resulting from inappropriate supervision and/or technique [90]. 

Growing research has shown yoga to be an effective therapy for chronic pain among adults, but studies remain limited for the pediatric population [91]. In a 2006 meta-analysis, when yoga was incorporated into school curricula, improvements in academic performance, behavior, concentration, emotional balance, and self-esteem were all seen [92]. Regular practice of yoga has been associated with improvement in mood and function in patients suffering from depression [93]. 

These same techniques can be of great benefit in the management of chronic pain in particular. One pilot study of a yoga program in pediatric patients with Irritable Bowel Syndrome (IBS) and functional abdominal pain, demonstrated significant decreases in pain frequency and intensity, as well as improved quality of life per parents report [94]. A study of 30 children, aged 11–18, with amplified pain syndromes enrolled in an intensive interdisciplinary pain rehabilitation program incorporating 5–6 h of yoga weekly demonstrated improved pain and functioning without the use of pharmacology therapy [95]. Positive effects have also been shown in pediatric patients with rheumatoid arthritis [96]. Current evidence is encouraging, but more large-scale research is still needed to determine the efficacy of yoga in the treatment of pediatric chronic pain [39]. 

#### 3.2.4. Acupuncture

Acupuncture has been a focus of Traditional Chinese Medicine (TCM) for thousands of years and since its introduction into western medicine, it has become one of the most popular complementary and alternative medical (CAM) therapies in the US [97]. First acknowledged as a medical therapy by the National Institute of Health (NIH) in 1997, the number of licensed acupuncturists in the US has grown exponentially over the decades with over 27,000 licensed acupuncturists in 2013—a one hundred percent increase from 2000—and an estimated one million annual American consumers. In 2001, Battlefield Acupuncture, an auricular acupuncture procedure, was developed by Richard Niemtzow as a simple and effective method for rapid pain relief that can be performed with minimal training by non-licensed acupuncturists [98,99,100]. Since then, it has since been utilized in emergency rooms and has been taught to US military and North Atlantic Treaty Organization (NATO) personnel for use in both medical and battlefield environments [99,101]. 

Research into the efficacy of the therapy has also increased over the years with many studies demonstrating acupuncture to be more effective at treating chronic pain than placebo, standard care, and no care [102,103,104,105,106,107,108,109,110,111,112]. Acupuncture is a viable alternative for the treatment and management of acute and chronic pain across various illnesses [22,103,113,114,115]. One overview of Cochrane reviews concluded that acupuncture demonstrates effectiveness as a treatment for pain associated with migraines, tension headaches, neck disorders, arthritis, and low back pain [104]. 

Research studies aiming at understanding the physiological and neurological mechanisms that make acupuncture an effective therapy have produced interesting results that have served as the basis for several potential hypotheses [107,116,117,118,119,120,121,122,123,124,125,126,127,128,129]. Many researchers believe that acupuncture stimulates nerves and muscles located within the acupuncture points and trigger a release of neurochemicals endorphins such as serotonin, oxytocin, and endogenous opioid peptides and therefore result in an analgesic effect [117,123,127]. However, while substantial research has shown acupuncture to be an effective therapy for pain among the adult population, there is limited research on acupuncture in regards to the treatment of pain among pediatric patients. Nonetheless, what little research does exist concludes that acupuncture is an effective and feasible therapy option for pain in pediatric populations [48,103,130,131,132,133,134]. One study in the pediatric intensive care unit (PICU) that used acupuncture for acute post-operative pain in children showed that acupuncture was highly accepted and well tolerated without morbidity. In addition, the majority (70%) of patients and families surveyed believed that acupuncture helped their pain. This particular study used the Japanese form of acupuncture with fine needles for older children and Shonishin (a non-needling technique using special tools) for those who were younger than two years of age [135]. A number of other articles have also shown some perceived pain relief without significant adverse effects due to acupuncture. [136,137]

Though acupuncture is safe when administered by appropriately trained and credentialed practitioners, there are some children who have a fear of needles or for medical reasons such as low platelet count or immunodeficiency that may not be recommended to receive acupuncture. For those patients, other techniques such as Shonishin or acupressure can be employed. 

## 4. Discussion: H.O.P.E, A New Paradigm

The use of mind–body techniques in pediatrics can be a powerful adjunct to empower patients and their families and to give them hope for a brighter future with improved quality of life. In partnership with their physicians, families can begin to uncover what is in their tool box for pain and symptom management. One such framework developed by us is the pneumonic: H.O.P.E. We have used H.O.P.E. with success in clinical practice with families as a strategy for approaching pain management. H.O.P.E. addresses four essential components in the evaluation of pain and of the treatment plan. It is described in more detail below.

- H: **How** has the pain affected you and impacted your quality of life? **How** have they addressed the pain in the past and what therapies have been used?
- O: **Observations** about previous management approaches. What have they **observed**? What worked and what did not? What makes the pain perception worse?
- P: **Plan** for the future, set goals and determine the treatment plan.
- E: **Evaluate** the **efficacy** of the plan and manage **expectations.**

In addition to understanding what pharmacologic, non-pharmacological and integrative options are available; it is also important for all parties involved to discuss goals of care. In fully unpacking the child and family’s needs, appropriate recommendations for pain management can be made. Recommendations will be different based on the clinical condition and goals of care.

## 5. Conclusions

Mind–body medicine provides an important approach to the management of both acute and chronic pain in the pediatric population. There are many modalities that are underutilized in pediatrics including mindfulness, hypnosis, yoga and acupuncture. It is important to note that these can be of significant benefit with minimal risk. Mind–body approaches can be used alongside conventional treatment or in some cases as the primary treatment for pain. Though more research is needed; the mind–body approaches discussed are recommended for patients who have no underlying contraindications. As our understanding of pediatric pain and pain management continues to mature, mind–body medicine is an area that is ripe for future studies and investigations.
